# Low-Density Particleboards Modified with Expanded and Unexpanded Fillers—Characteristics and Properties

**DOI:** 10.3390/ma15134430

**Published:** 2022-06-23

**Authors:** Piotr Boruszewski, Piotr Borysiuk, Agnieszka Jankowska, Jolanta Pazik

**Affiliations:** 1 Institute of Wood Sciences and Furniture, Warsaw University of Life Sciences-SGGW, 159 Nowoursynowska St., 02-776 Warsaw, Poland; piotr_borysiuk@sggw.edu.pl (P.B.); agnieszka_jankowska@sggw.edu.pl (A.J.); 2 Fabryki Mebli “FORTE” S.A., 1 Biała St., 07-300 Ostrów Mazowiecka, Poland; jolanta.pazik@forte.com.pl

**Keywords:** raw material, low-density particleboard, expanded and unexpanded filler, particleboard, physical and mechanical properties, foamed polymers

## Abstract

Reducing the density of wood-based materials is a desirable research direction in the development of the wood-based materials sector. Even though lightweight wooden particleboards have been commercially available for many years, they still have a number of disadvantages, especially their low strength parameters. The aim of this paper was to determine the possibility of producing particleboards of reduced density for use in the furniture industry, as a result of using expanded polystyrene and two types of microspheres (expanded and unexpanded) to modify the core layer of three-layer particleboards. Analysis of the results of testing the particleboards’ properties when using various types of modifiers (expanded and unexpanded fillers), urea formaldehyde (UF) glue content (high: 10%/12% and low: 8%/10%), various glue-dosing methods, and different particle sizes, allows us to conclude that the most satisfactory effect was found when using EPS. One partly positive effect was observed when using the Expancel-type 031 DU 40 as a filler; therefore, it is recommended that research be continued in this area. Using microspheres that have not been used before as a filler in the production of wood-based panels is the novelty of the presented research. The proposed technology has potential for application in the industry.

## 1. Introduction

Wood materials with reduced density, intended for applications in the furniture industry, are now a desirable research direction in the development of the wood-based materials sector. Their main advantage is the reduced unit weight, resulting in the reduced mass of the final product made from these materials (e.g., furniture). Other favorable features include the possibility of reducing the quantity of wood needed as raw material and other components in the produced boards, as well as facilitating the transport of lightweight boards and reducing the costs [1]. The reduction of basic raw materials also translates directly into the possibility of reducing the emission of harmful volatile organic compounds (especially formaldehyde), making it possible to manufacture wood-based materials with low volatile organic compound (VOC) emissions (ultimately reaching the Super E-Zero class). Limiting the consumption of wood also prompts the search for complementary raw materials that could be used in particleboard production, particularly low-density particleboards. The by-products of agricultural activity and agri-food processing are potential sources of raw material [2]. Possibilities for application in the wood-based panel industry have included, among others, such waste products of the agricultural industry as cereal straw [3,4,5,6], cotton, hemp and jute stalks [7,8], rape straw [9], miscanthus and flax [10], bagasse [11], elements of corn waste [12,13], pineapple leaves [14], tomato stalks [15], eggplant stalks [16], vine prunings [17], and sugar beet pulp [18]. The literature also presents the results of research on the use of other alternative materials, such as expanded materials/expanded polystyrene or expanded corn [19,20,21], waste polyurethane foam [22], and wastepaper [23,24].

Currently, several varieties of furniture boards with reduced density are available on the European market. These are mainly cellular boards in which the middle layer is made in the form of an openwork structure, e.g., honeycomb paper, an arrangement of upright hardboards or HDF, and Dendrolight material [25,26,27,28]. A particular limitation in the use of the aforementioned types of cellular boards is their internal “empty” structure, which requires the use of specialized furniture hardware systems. This problem can be overcome in the case of lightweight particleboards with a modified core layer containing special fillers [29]. The lack of modification of the surface layers of particleboards with reduced density will avoid limitations in terms of finishing their surface (applying foil, laminates, etc.). In recent years, taking into account the latest reports, a number of experiments on the possibility of lowering the density of wood-based panels have been made [30,31,32,33]. The proposed changes often include the diversification of the lignocellulosic raw materials used, mainly in terms of the use of low-density raw materials, e.g., poplar wood, fast-growing wood, and lignocellulosic particles from biomass obtained in an annual cycle [12,34,35,36,37]. Moreover, modifications of the technological parameters of the production process are proposed, mainly with regard to changes in the pressing curve [38] with the simultaneous injection of steam for a period of approx. 20% of the total pressing time [39]. Some attempts were also made to introduce modifiers to the board structure, reducing the consumption of wood raw material and, at the same time, obtaining a porous board structure as a result of reducing the density [19,22,40]. It turns out, however, that the economic aspects of the presented solutions negatively affect their widespread implementation in industrial practice. In summary, it can be stated that the particleboards with reduced density are an interesting material for both the furniture industry and the board industry. On the one hand, they allow designers to reduce the weight of the furniture, and on the other hand, they allow for savings in terms of resources and the energy needed for their production.

Foaming the polymer contained in the composite is an effective procedure that significantly reduces the density and extends the scope of the use of wood-based panels. In the context of particleboard, the “foaming” of the polymer can refer to both the foaming of the binder joining the wood particles and the production of boards with a foam-type core. In the first case, there is a reduction in the binder content of the final product, while in the second case, there is a partial replacement of both the binder and the lignocellulosic material with polymer foam. Thanks to this solution, products with a more uniform density in cross-section are obtained; this makes them easier to join with metal connectors. Wood composites with foams also show better impact strength [41], favorable price–quality performance, and strength-to-weight ratio [42]. Due to the plasticizing effect of the gas used, the production of such products takes place at a lower temperature and is faster than in the case of non-foamed products, which reduces the costs of the process [43]. The first batch process for the production of microporous composites was presented by Martini et al. [44], followed by the introduction of continuous extrusion, injection, and pressure molding system.

The objective of the present paper is to determine the possibility of producing particleboards with a density of 520 kg/m^3^ (suitable for the furniture industry) as a result of using expanded polystyrene and two types of microspheres (expanded and unexpanded), which are added to the adhesive resin used for bonding the particles of the core layer of the particleboard. The suitability of the particleboards for furniture manufacturing was determined by assessing selected mechanical and physical properties that are important in the production and use of furniture. So far, microspheres have been used as a lightweight filler in thermosets, adhesives, underbody coatings, and similar applications. Until now, they have not been used as fillers for the production of wood-based panels.

## 2. Materials and Methods

### 2.1. Particleboards Manufacturing

The research assumed the production of three-layer particleboards with a density that was reduced to 520 kg/m^3^. The dimensions of the length, width, and thickness of the boards were: 320 × 320 × 15 mm^3^. Boards were produced in 16 variants, with 10 repetitions each. The characteristics of the assumptions of the individual variants within which three-layer particleboards were produced are presented in Table 1.

The assumptions of the adopted research plan (Table 1) were based on the Taguchi method, which allows obtaining highly reliable research results while eliminating those factors that are difficult or even impossible to control (the so-called disturbing factors that negatively affect the final result). The production of particleboards within individual variants was differentiated by four factors at different levels of variability, i.e.: type of filler—4 levels of variation, glue content—2 levels of variation, glue dosing—2 levels of variability, particle size of the core layer—2 levels of variability.

Industrial Scots pine (*Pinus sylvestris* L.) particles were used for the production of boards with an average particle moisture content of 4.8% in the core layer and an average particle moisture content in the face layers of 4.3%. As part of the experiment, it was planned that core layer particles of different geometries were used for the production of boards (thickness of 0.4 mm and various lengths of 8 mm and 4 mm), while the face layer particles were typical of those used in industrial production.

Before forming the mats, the wood particles were bonded with industrial urea-formaldehyde (UF) resin (AB Achema, Jonavos, Lithuania) at a concentration of 65%, hardened with a 10% aqueous solution of ammonium sulfate (Merck KGaA, Darmstadt, Germany). UF resin is a standard binder used in the production of particleboards. The dry hardener content, in relation to the dry weight of the adhesive resin, was 0.2% (both in the case of an adhesive recipe dedicated to bonding the particles of the face layers as well as the particles of the core layer). The boards manufactured according to the assumptions of variants V to XVI were modified (Table 1). The modification consisted in introducing to the adhesive resin used for bonding the particles intended for the core layer of fillers (in the form of expanded and unexpanded microspheres with closed structures, allowing obtaining a resin with a reduced density) in the amount of 1% in relation to the weight of the adhesive resin, with a concentration of 65%. The following were used as fillers:

- expanded microspheres, made of polymethyl methacrylate (PMMA)—Expancel type 920 DE 40 d30 (Nouryon B.V., Amsterdam, The Netherlands);
- unexpanded microspheres, increasing their volume under the influence of increased temperature—Expancel type 031 DU 40 (Nouryon B.V., Amsterdam, The Netherlands);
- expanded polystyrene EPS (Unipol Holland BV, Oss, The Netherlands).

The range of expansion of the above-mentioned microspheres was within the range of processing of the UF resin. In the production of the boards, a hydrophobic agent was also used in the form of a paraffin emulsion (Polwax S.A., Jasło, Poland) in an amount of 1% relative to the dry weight of the particles.

In the next stage, bonded particles were used to prepare a three-layer mat that was hand-formed. The formed mats were pre-cold-pressed at a pressure of 0.5 MPa for 30 s. The main pressing was carried out with a computer-controlled press. The pressing parameters were selected on the basis of industrial conditions and data presented in the literature [45]: the maximum unit pressure was 2.5 MPa (this was maintained until the required board thickness was achieved, then successively reduced until the end of the assumed time of pressing); the press plate temperature was 180 °C; the pressing factor was 18 s/mm; the press closing speed was 2 mm/s, and the total pressing time was 270 s. The individual pressing parameters were measured automatically, with the following accuracy: temperature of the mat core, ± 0.01 °C, pressure ± 0.01 MPa, and the thickness of the mat, ±0.01 mm. The temperature inside the mat was measured with a Fe-CuNi thermocouple fixed into the mat’s core during its formation.

### 2.2. Particleboard Properties

Before the samples were prepared for the determination of individual properties (in accordance with the relevant standards), the boards were first calibrated by grinding their surface. In the next stage, the samples were conditioned until a constant weight was obtained (climate conditions: relative air humidity 65%, air temperature 20 °C).

The first properties test determined the density of the board samples, based on the assumptions of the EN 323: 1999 standard [46]. The change in density profile was determined from a cross-section of the boards using a laboratory X-ray density analyzer GreCon Da-X (Fagus-Grecon Greten GmbH & Co. KG, Alfeld-Hannover, Germany). Measurements were made with a scanning accuracy of 0.02 mm and a sample speed of 0.05 mm/s.

Based on the assumptions of the EN 310: 1993 standard [47], the static bending strength (MOR) of the boards and their modulus of elasticity in static bending (MOE) was determined. The tensile strength test, perpendicular to the board plane (IB), was carried out in accordance with the guidelines of the EN 319: 1993 standard [48]. Based on the assumptions of the EN 320: 2011 standard [49], a test to determine the force required to pull a screw (holding capacity of the screw) out of the tested particleboards perpendicular to the surface (SH Ʇ) and parallel to the surface (SH II) was carried out. The surface hardness (HB) was determined based on the EN 1534: 2020-06 standard [50]. The swelling thickness of the boards was determined after 24 h of soaking the samples in water, in accordance with the requirements of the EN 317: 1999 standard [51].

At least 10 repetitions were performed for each of the determined properties of the tested boards. The mean values of tested parameters were compared using a one-way analysis of variance (ANOVA) and Tukey’s post hoc test, in which homogeneous groups of mean values for each parameter were identified for *p* = 0.05. The significance of the influence on the considered variables was calculated using a multi-factor ANOVA test by the determination of percentage contribution for the analyzed factors. The experimental data were statistically analyzed using the STATISTICA 13.3 software (TIBCO Software Inc., Palo Alto, CA, USA).

## 3. Results and Discussion

### 3.1. Density Profile

**Figure 1:**
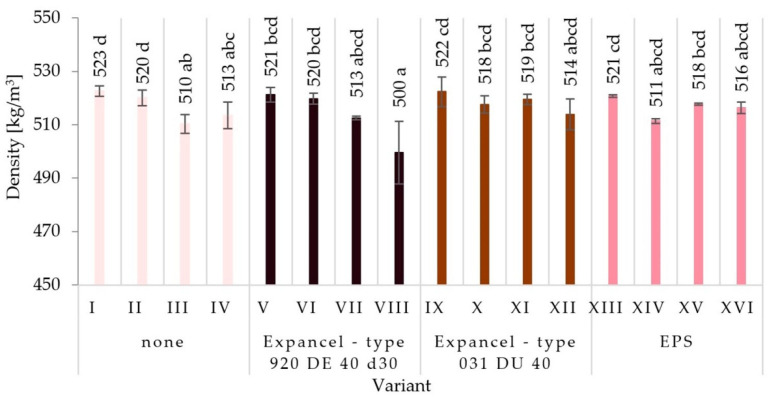
The density of the particleboards produced (means and standard deviation, a, b, c, (…). Homogeneous groups were determined by the Tukey test; different letters denote a significant difference; means followed by the same letter do not statistically differ from each other).

The results of the measurement of particleboard density differing in the type of filler used, the glue content level, the rate of glue dosing, and the size of the particles used are summarized in Figure 1. The highest value of density was recorded for variant I (523 kg/m^3)^, and the lowest for variant VIII (500 kg/m^3^). The noted changes in the values of the average density of the boards (made in accordance with the assumptions of individual variants) should not have a significant impact in terms of differences in the values of the analyzed properties of the boards.

**Figure 2:**
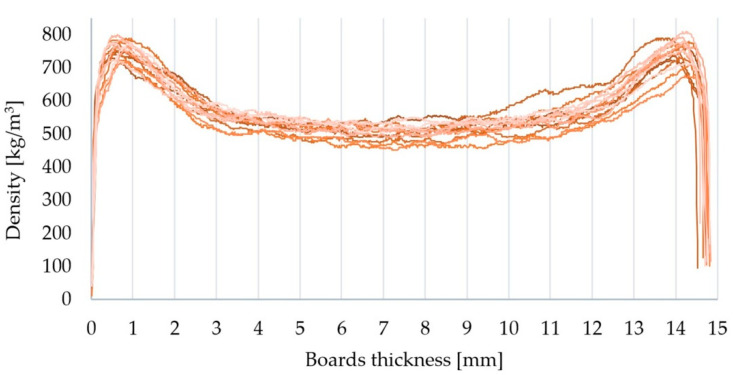
Density profiles of the particleboards produced.

The courses of the density profiles on the cross-section obtained for the manufactured particleboards are summarized in Figure 2. All of them were characterized by a similar, typically U-shaped, and symmetrical path. This confirms the correctness of their manufacture. The density of the particleboard is not uniform at the cross-section; most often, it takes a U-shaped course. This change occurred as a result of the pressing process, through the direct effects of heat and pressure on the particleboard. The obtained density profile on the cross-section depends on a number of factors, including the press closing speed, moisture distribution in the mat, and the temperature of the hot press plates [52,53]. The arrangement of the particles in the mat, the type of wood used for particleboard production, and the type of resin used to bond the particles also influence the course of the particleboard density profile. The analysis of the obtained particleboard density profile on the cross-section is used to predict the values for certain properties of the boards, including static bending strength (MOR), modulus of elasticity in static bending (MOE), internal bond strength (IB), screw-holding capacity and dimensional stability [54,55,56,57]. Wong et al. [55,56] reported that a typical particleboard with a U-shaped course in the density profile was of higher MOR and MOE values compared to boards made of a homogeneous material with the same average density. The higher density of the surface layers increased the obtained MOR and MOE values. The opposite phenomenon was observed regarding the IB value, due to the lower core layer density of conventional particleboard. Hence, with regard to the final application of particleboards, it is important to properly control the parameters of the pressing process in order to obtain an appropriate density profile in the cross-section.

### 3.2. Mechanical Properties

**Figure 3:**
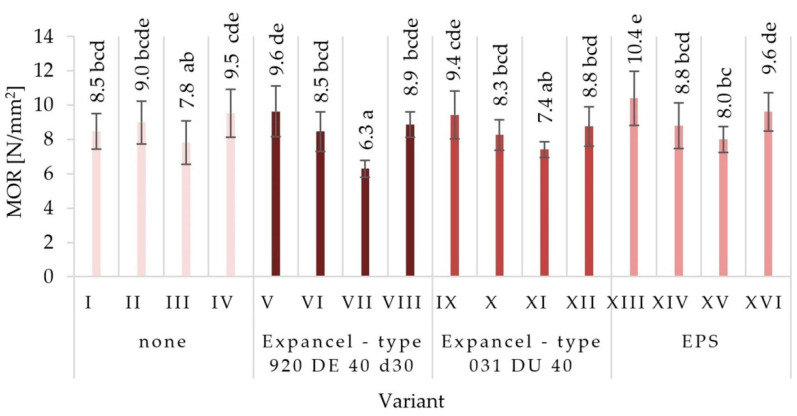
Static bending strength (MOR) of the particleboards produced (means and standard deviation, a, b, c, (…). Homogeneous groups were determined by the Tukey test; different letters denote a significant difference; means followed by the same letter do not statistically differ from each other).

**Figure 4:**
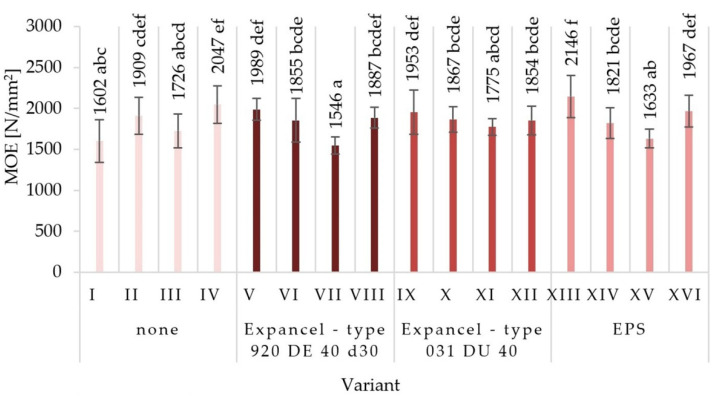
Modulus of elasticity (MOE) of the particleboards produced (means and standard deviation, a, b, c, (…). Homogeneous groups were determined by the Tukey test; different letters denote a significant difference; means followed by the same letter do not statistically differ from each other).

**Figure 5:**
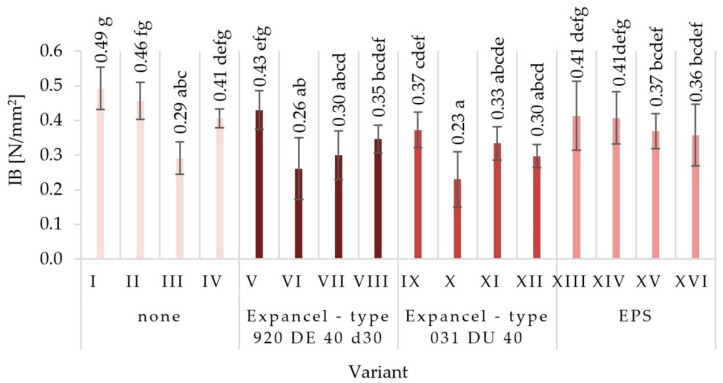
Internal bond strength (IB) of the particleboards produced (means and standard deviation, a, b, c, (…). Homogeneous groups were determined by the Tukey test; different letters denote a significant difference; means followed by the same letter do not statistically differ from each other).

**Figure 6:**
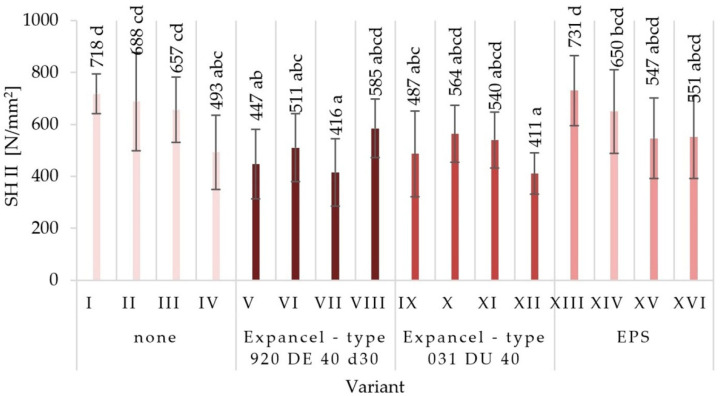
Screw-holding capacity parallel to the surface (SH II) of the particleboards produced (means and standard deviation, a, b, c, (…). Homogeneous groups were determined by the Tukey test; different letters denote a significant difference; means followed by the same letter do not statistically differ from each other).

**Figure 7:**
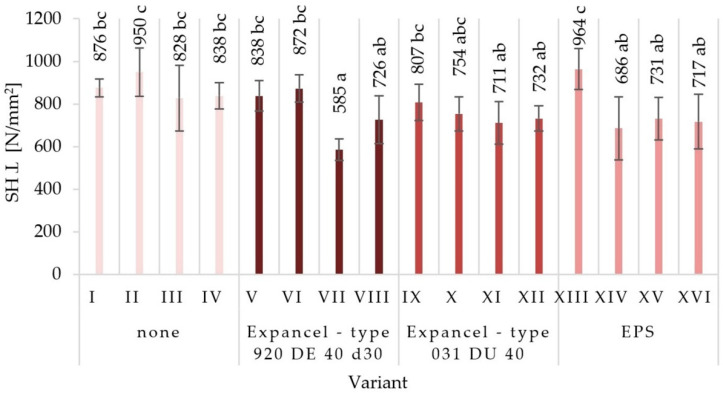
Screw-holding capacity perpendicular to the surface (SH Ʇ) of the particleboards produced (means and standard deviation, a, b, c, (…). Homogeneous groups were determined by the Tukey test; different letters denote a significant difference; means followed by the same letter do not statistically differ from each other).

**Figure 8:**
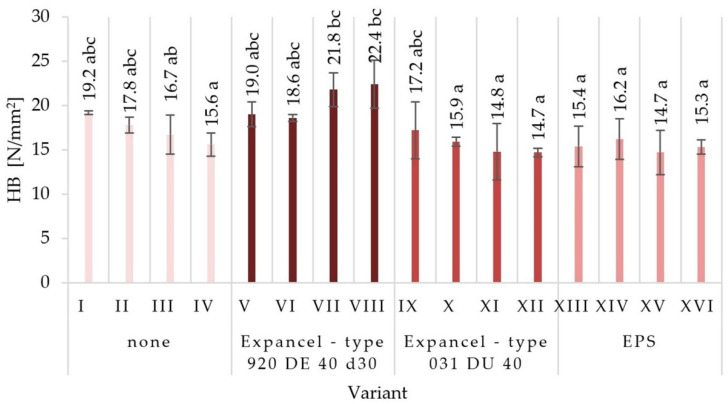
Surface hardness (HB) of the particleboards produced (means and standard deviation, a, b, c, (…). Homogeneous groups were determined by the Tukey test; different letters denote a significant difference; means followed by the same letter do not statistically differ from each other).

The average values of mechanical properties for those particleboards made under the assumptions of individual variants were obtained together with the values of standard deviations in Figure 3, Figure 4, Figure 5, Figure 6, Figure 7 and Figure 8. The highest value of static bending strength (MOR) was characteristic for variant XIII (10.4 N/mm^2^). The boards produced under the assumptions of variants IV, V, and XVI were also characterized by high values of MOR, these being 9.5 N/mm^2^, 9.6 N/mm^2^, and 9.6 N/mm^2^, respectively. The lowest values of the determined properties were obtained for variants VII (6.3 N/mm^2^), XI (7.4 N/mm^2^), and III (7.8 N/mm^2^). With regard to the determination of the modulus of elasticity in static bending (MOE), the highest value was obtained for variant XIII (2146 N/mm^2^) and the lowest one for variant VII (1546 N/mm^2^). Comparing the obtained values for the internal bond (IB), it was found that the highest values were seen in the boards from variants I, II, and V (0.49 N/mm^2^, 0.46 N/mm^2^, and 0.43 N/mm^2^, respectively), while the lowest values of the internal bond were characterized for variants X (0.23 N/mm^2^) and VI (0.26 N/mm^2^). The panels made from variant XIII were distinguished by the highest screw-holding capacity value (964 N/mm^2^), while the boards made from variant II were comparable (950 N/mm^2^), and the lowest value was obtained for boards made from variant VII (585 N/mm^2^).

There were no significant differences between the values of the surface hardness of the individual variants of the produced particleboard. The lowest surface hardness (14.7 N/mm^2^) was characterized by variant XV, produced using EPS, with a glue content of 8%/10%. A very similar value of surface hardness was seen in the variant XII samples, characterized by 8%/10% of glue content, made using Expancel-type 031 DU 40. The highest surface hardness (22.4 N/mm^2^) was achieved for variant VIII boards produced using Expancel-type 920 DE 40 d30. The hardness of the surface depends mainly on the properties of the surface layers, which were not subject to material modification by introducing the fillers. However, the fillers used may affect the elasticity of the core layer of the particleboards, which in turn may affect the obtained values for surface hardness. The lowest hardness values were recorded for variants in which the EPS filler was used, and the highest ones for those in which Expancel-type 920 DE 40 d30 was used. The observed mean values of mechanical properties established via Tukey’s test were classified into different homogenous groups. The results of the statistical analysis provide the basis for selecting a compilation of the variables that give the most favorable results. Among the fillers used, EPS had the most favorable influence on the mechanical properties of particleboards (partly also Expancel-type 031 DU 40; therefore, it is recommended to continue research into this filler). At the same time, higher values for static bending strength, the modulus of elasticity, tensile strength, and the ability to hold onto the screws were achieved by using pneumatic spraying and a low glue content.

The obtained results from investigating the mechanical properties of particleboards led to the general conclusion that the greatest impact on the tested properties was mainly due to the increase in glue content and the type of glue dosing, and also partly due to the addition of EPS filler to the glue. There was no clear effect in terms of an increase in the strength parameters of the boards or the addition of the Expancel-type 920 DE 40 d30 and Expancel-type 031 DU 40 filler to the adhesive resin. However, the observed trends lead to a general conclusion that it would be justified to continue research in this area. Dunky [58] also indicated the significance of the glue content influence and the quality of its distribution on the surface of the particles, as well as the significance of the total surface of the particles coated with glue on the obtained properties of particleboards. When analyzing the obtained test results, it should be stated that in this study, there was no clear effect of the change in particle size of the internal layers on the strength parameters. As it is known that the geometry of the particles mainly affects the quality of their mutual bonding with glue joints, and among the various dimensions of the particle, their thickness and length are the most important [59,60]. The study of the effect of particle size in the outer layers of particleboards on the board’s bending strength showed that an increase in particle size causes a decrease in strength. The explanation for this is the decrease in the joining areas between the particles, resulting from their lower compaction [60].

The research results obtained in this study partially overlap with the observations presented in other studies [20,61,62,63]. Technical specification CEN/TS 16368: 2014 (Lightweight Particleboards—Specifications) presents the requirements for the specified mechanical properties of general-purpose lightweight boards, LP2, for use in dry conditions. According to CEN/TS 16368: 2014, for boards with a thickness range of >13 to 20, the MOR should be at least 7.0 N/mm^2^, the MOE should be at least 950 N/mm^2^, and the IB should be at least 0.35 N/mm^2^ [64]. The results indicate that these requirements were met for the boards manufactured in this study. It was reported that by including an EPS fraction in the core layer of the particleboard, a significant improvement in the mechanical properties (MOR, MOE, IB) of low-density particleboard can be obtained. Luo et al. [63] introduced much larger amounts of EPS into the core layer of the particleboards than resulted from the assumptions of the present study. Moreover, the influence of the mat pressing temperature share on the final properties of the panels was determined. Luo et al. [63] obtained a significant increase in internal bond strength due to the addition of EPS to the core layer of the particleboard. The EPS in the core of the board filled the voids between the wood particles, which allowed better integrity of the core layer and greater cohesive strength and, thus, increased IB strength. Moreover, it was shown that the higher pressing temperature negatively influenced the IB strength, especially with a high EPS content (10% and 12.5%). This phenomenon is in line with a previous study by Mir et al. [62], who found that the rise in press shelf temperature had a negative effect on the IB of lightweight particleboard using EPS as filler. However, Shalbafan et al. [20] reported that the use of foam fillers has a significant effect on MOR, MOE, screw-holding capacity parallel to the surface, and the thickness of swelling. On the other hand, the use of expandable filler did not affect the surface stability and screw-holding capacity perpendicular to the surface, because this mainly depends on the quality and density of the top layer. It is also reported that the physical and mechanical properties of the boards did not change radically when the amount of expandable filler was increased from 5 to 15%.

An increase in strength while maintaining low compression of the particleboards is possible to achieve by filling the existing voids in the board structure. The pores can be filled by using an additional component, e.g., expanded synthetic material in the form of granules such as EPS [19,40,65]. However, the indicated method of reducing the density of the boards may increase the production costs and extend the production process with additional operations. These additional operations concern the preparation and application of filler to the wooden particles of the inner layer of particleboards, along with their homogenization.

Considering the percentage contribution to the influence of individual variable factors on the strength properties of the boards, it should be stated that fillers and glue dosing played the most important role (Table 2). The fillers were most affected in the case of HB (*P* = 53.66%). With reference to IB, SH II, and SH Ʇ, the effect of fillers is *P* = 20.04%, 17.73% and 14.57%, respectively. Only in the case of MOE, fillers did not show a statistically significant effect (*P* = 1.21%). Glue dosing had the greatest effect on MOR, MOE, and IB (*P* = 20.88%, 15.06%, and 10.61%, respectively). In the case of other mechanical properties, the influence of this factor was statistically insignificant. Among the examined factors, the particle size showed the least influence (*P* from 0.30% to 7.42%). It should be noted that in relation to MOR, MOE, and SH Ʇ, this effect is statistically significant. The total effect of the tested factors on the strength properties of the boards (except in the case of HB) was smaller than the influence of the factors not included in these tests. Depending on the tested features, the error values ranged from 56.34% to 76.34%.

### 3.3. Swelling Thickness

**Figure 9:**
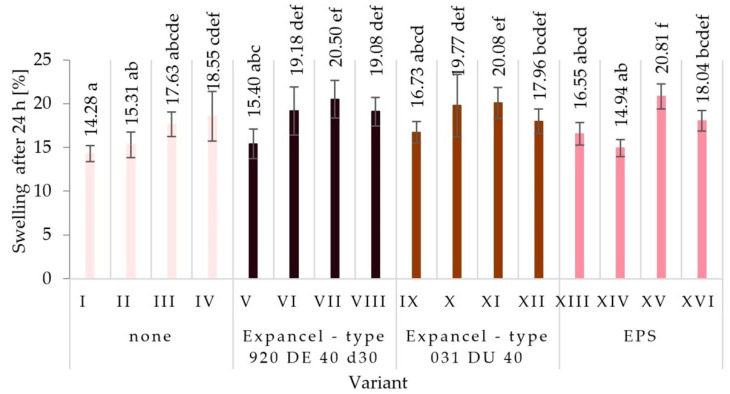
Swelling thickness changes of the produced particleboards after 24 h in water (means and standard deviation, a, b, c, (…). Homogeneous groups were determined by the Tukey test; different letters denote a significant difference; means followed by the same letter do not statistically differ from each other).

Analysis of the test results of swelling thickness after soaking the particleboard samples in water for 24 h allows for the observation of differences in the dimensional stability of the particleboards produced according to the assumptions of the individual variants. The results of the average swelling thickness values for individual variants of particleboards are presented in Figure 9. Variant I (14.3%) was characterized by the highest dimensional stability, with the smallest range of changes in swelling thickness after 24 h of soaking the samples in water. Variants II, V, and XIV showed an equally small range of changes (15.3%, 15.4%, and 14.9%, respectively). The highest value of swelling thickness was found in variant XV (20.8%). High values of swelling thickness were also noted for variants VII and XI (20.5% and 20.1%, respectively). The obtained results were in line with expectations. It is more favorable for dimensional stability to use a higher glue content level. It has been shown that the dosing of glue on the particles using pneumatic spraying has a positive effect on the board’s dimensional stability (a lower value of swelling thickness after 24 h of soaking the samples in water). In general, the use of tested fillers does not affect the dimensional stability of the tested boards.

The thickness swelling of the particleboards is related both to their internal structure (porosity) and the raw materials used to produce them [66]. On the one hand, reducing the density of the boards (increasing the porosity) facilitates the penetration of moisture into the board; on the other hand, increasing the porosity reduces the swelling of the boards due to the lower number of lignocellulosic particles [67,68]. Shalbafan et al. [20] reported that introducing filler in the form of EPS granules into the voids in the board reduces the penetration of moisture and, consequently, the swelling of the boards. Polystyrene is a hydrophobic material; it does not absorb moisture and does not change its dimensions under the influence of moisture [69]. When examining the effect of adding granulate EPS to particleboards, Dziurka et al. [61] found that the reduction in swelling thickness of the boards is due to the more porous structure of the board, rather than to partially replacing the middle layer particles with highly hydrophobic polystyrene granules. The type of filler has not been shown to influence the decrease in swelling thickness, which may be related to the aforementioned influence of the porous structure of the boards. However, the presence of hydrophobic filler particles may limit the distribution of the particle adhesive. It is generally assumed that an increase in the degree of gluing translates into a decrease in plate swelling [66]. This is also confirmed in the presented research, but the method of distributing the adhesive on the particle also played an important role in this respect.

When analyzing the percentage contribution to the influence of individual examined factors on the thickness swelling of the boards (Table 3), the greatest influence was noted for glue content and kind of filler (*P* = 25.87% and 10.35%, respectively). The glue dosing and particle size influence contribution were *P* = 6.66% and 3.82%, respectively. The influence of all factors was statistically significant. It should be noted that, as in the case of the strength properties, as well as swelling thickness, the total influence of the examined factors is smaller than the influence of factors not included in this study (error = 53.29%). Factors that could have influenced the swelling that were not determined may include the porosity of the particleboards.

## 4. Conclusions

Based on our analysis of the results of testing the particleboards properties with various types of modifiers (fillers), degrees of gluing (high: 12%/10% and low: 10%/8%), glue dosing methods (flow-dosing and pneumatic spraying), and different particle sizes, the following conclusions can be drawn:

1. The addition of expanded polystyrene EPS (and to a lesser extent, Expancel-type 920 DE 40 d30) as a filler had a positive effect on the mechanical properties of the three-layer particleboards. Variant XIII (made with EPS) was characterized by the highest value of static bending strength. The highest value of tensile strength perpendicular to the planes was demonstrated by particleboards with fillers made from variants V (made with Expancel-type 920 DE 40 d30), XIII, and XIV (made with EPS). Particleboards from variant XIII (made with EPS) were distinguished by the highest resistance values when pulling out the screws axially.
2. EPS had the most important influence on the mechanical properties of particleboards (also, partly, Expancel-type 031 DU 40, therefore it is recommended that research should continue with the inclusion of this filler). At the same time, higher values of static bending strength, modulus of elasticity, tensile strength, and the ability to hold onto the screws were achieved using pneumatic spraying and a lower glue content.
3. The addition of EPS as the filler had a positive effect on dimensional stability. The smallest range of dimensional changes after 24 h of soaking in water was characteristic for particleboards from variant XIV, with a high degree of gluing, flow-dosing of the glue, and the use of larger particles (made with the participation of EPS).
4. Both expanded and unexpanded fillers allow for the production of particleboards with reduced density and thus lead to savings in terms of wood raw material and, consequently, a reduction in the share of adhesive resins, which are largely responsible for the volatile organic compound emissions from the boards. Further research into the use of microspheres may also increase the percentage of fillers.

## Figures and Tables

**Table 1:** Characteristics of individual variants, as part of which three-layer particleboards were produced.

Variant	Type of Filler	Glue Content of the Core/Surface Layer (%)	Glue Dosing	Length of Core Layer Particles (mm)
**I**	-	10/12	pneumatic spraying	8
**II**	-	10/12	flow dosing	4
**III**	-	8/10	flow dosing	8
**IV**	-	8/10	pneumatic spraying	4
**V**	Expancel-type 920 DE 40 d30 *	10/12	pneumatic spraying	8
**VI**	Expancel-type 920 DE 40 d30	10/12	flow dosing	4
**VII**	Expancel-type 920 DE 40 d30	8/10	flow dosing	8
**VIII**	Expancel-type 920 DE 40 d30	8/10	pneumatic spraying	4
**IX**	Expancel-type 031 DU 40 *	10/12	pneumatic spraying	4
**X**	Expancel-type 031 DU 40	10/12	flow dosing	8
**XI**	Expancel-type 031 DU 40	8/10	flow dosing	4
**XII**	Expancel-type 031 DU 40	8/10	pneumatic spraying	8
**XIII**	EPS	10/12	pneumatic spraying	4
**XIV**	EPS	10/12	flow dosing	8
**XV**	EPS	8/10	flow dosing	4
**XVI**	EPS	8/10	pneumatic spraying	8

Note: * commercial designations.

**Table 2:** ANOVA for selected factors affecting the MOR, MOE, IB, SH, and HB of the manufactured particleboards (*p* = probability of non-significant effects, *P* = percentage influence).

Source of Variation	MOR		MOE		IB		SH II		SH Ʇ		HB	
	*p*	*P* (%)	*p*	*P* (%)	*p*	*P* (%)	*p*	*P* (%)	*p*	*P* (%)	*p*	*P* (%)
**Filler**	0.002	5.32	0.417	1.21	0.000	20.04	0.000	17.73	0.001	14.57	0.000	53.66
**Glue content**	0.000	6.98	0.000	5.35	0.001	6.14	0.002	5.06	0.000	18.62	0.490	0.53
**Particle size**	0.014	2.16	0.011	2.77	0.123	1.27	0.330	0.48	0.003	7.42	0.604	0.30
**Glue dosing**	0.000	20.88	0.000	15.06	0.000	10.61	0.383	0.39	0.052	3.05	0.624	0.27
**Error**		64.66		75.62		61.94		76.34		56.34		45.24

**Table 3:** ANOVA for selected factors affecting the TS of the manufactured particleboards (*p* = probability of non-significant effects, *P* = percentage influence).

Source of Variation	TS	
	*p*	*P* (%)
**Filler**	0.000	10.35
**Glue content**	0.000	25.87
**Particle size**	0.005	3.82
**Glue dosing**	0.000	6.66
**Error**		53.29

## Data Availability

Not applicable.

## References

[1] Thomen H. Lightweight panels for the European furniture industry: Some recent developments. Proceedings of the COST E49 Workshop on “Lightweight Wood based Composites”.

[2] Thole V. (2001). Faserplatten aus Palmenresten. Holz Kunstst..

[3] Boquillon N., Elbez G., Schönfeld U. (2004). Properties of wheat straw particleboards bonded with different types of resin. J. Wood Sci..

[4] Zhang Y., Lu X., Pizzi A., Delmotte L. (2003). Wheat straw particleboard bonding improvements by enzyme pretreatment. Holz als Roh- und Werkst..

[5] Dai C., Wasylciw W., Jin J. (2004). Comparison of the pressing behaviour of wood particleboard and strawboard. Wood Sci. Technol..

[6] Mo X., Cheng E., Wang D., Sun X. (2003). Physical properties of medium-density wheat straw particleboard using different adhesives. Ind. Crop. Prod..

[7] Guler C., Ozen R. (2004). Some properties of particleboards made from cotton stalks (*Gossypium hirsitum* L.). Holz als Roh- und Werkst..

[8] Alma M.H., Kalaycıoğlu H., Bektaş I., Tutus A. (2005). Properties of cotton carpel-based particleboards. Ind. Crop. Prod..

[9] Dziurka D., Mirski R. (2013). Lightweight boards from wood and rape straw particles. Drewno.

[10] Tröger F., Wegener G., Seemann C. (1998). Miscanthus and flax as raw material for reinforced particleboards. Ind. Crop. Prod..

[11] Ghalehno M.D., Nazerian M., Bayatkashkooli A. (2010). Influence of utilization of bagasse in surface layer on bending strength of three-layer particleboard. Holz als Roh- und Werkst..

[12] Wang D., Sun X.S. (2002). Low density particleboard from wheat straw and corn pith. Ind. Crop. Prod..

[13] Sekaluvu L., Tumutegyereize P., Kiggundu N. (2013). Investigation of Factors Affecting the Production and Properties of Maize Cob-Particleboards. Waste Biomass-Valorization.

[14] Tangjuank S. (2011). Thermal insulation and physical properties of particleboards from pineapple leaves. Int. J. Phys. Sci..

[15] Guuntekin E., Uner B., Karakus B. (2009). Chemical composition of tomato (*Solanum lycopersicum*) stalk and suitability in the particleboard production. J. Environ. Biol..

[16] Guntekin E., Karakus B. (2008). Feasibility of using eggplant (*Solanum melongena*) stalks in the production of experimental particleboard. Ind. Crop. Prod..

[17] Ntalos G.A., Grigoriou A.H. (2002). Characterization and utilisation of vine prunings as a wood substitute for particleboard production. Ind. Crop. Prod..

[18] Borysiuk P., Jenczyk-Tolloczko I., Auriga R., Kordzikowski M. (2019). Sugar beet pulp as raw material for particleboard production. Ind. Crop. Prod..

[19] Borysiuk P., Szołucha M., Jaskółowski W., Czechowska J. (2010). Low-density particleboards with foamed polystyrene additive. Ann Wars Univ Life Sci-SGGW. For. Wood Technol..

[20] Shalbafan A., Tackmann O., Welling J. (2015). Using of expandable fillers to produce low density particleboard. Holz als Roh- und Werkst..

[21] Lu J., Wang D., Jiang P., Zhang S., Chen Z., Bourbigot S., Fontaine G., Wei M. (2021). Design of fire resistant, sound-absorbing and thermal-insulated expandable polystyrene based lightweight particleboard composites. Constr. Build. Mater..

[22] Czechowska J., Borysiuk P., Mamiński M., Boruszewski P. (2008). Low-density particleboards filled with waste PUR foam. Ann. Wars Univ. Life Sci-SGGW. For. Wood Technol..

[23] Okino E.Y.A., Santana M.A.E., de Souza M.R. (2000). Utilization of wastepaper to manufacture low density boards. Bioresour. Technol..

[24] Taramian A., Doosthoseini K., Mirshokraii S.A., Faezipour M. (2007). Particleboard manufacturing: An innovative way to recycle paper sludge. Waste Manag..

[25] Luedtke J., Thoemen H., Welling J., Barbu C.M. (2008). Lightweight Wood-Based Board and Process for Producing It. Worldwide Patent.

[26] Jarusombuti S., Hiziroglu S., Bauchongkol P., Fueangvivat V. (2009). Properties of Sandwich-Type Panels Made from Bamboo and Rice Straw. For. Prod. J..

[27] Chen Z., Yan N., Deng J., Smith G. (2011). Flexural creep behavior of sandwich panels containing Kraft paper honeycomb core and wood composite skins. Mater. Sci. Eng. A.

[28] Shalbafan A., Luedtke J., Welling J., Thoemen H. (2011). Comparison of foam core materials in innovative lightweight wood-based panels. Holz als Roh- und Werkst..

[29] Ritter N., Kharazipour A. (2009). Development of three-layered popcorn based particleboards by a combinition of maize and wood. Review of Forests, Wood Products and Wood Biotechnology of Iran and Germany—Part III, Universitätsverlag Göttingen.

[30] Niemz P., Sandberg D. (2022). Critical wood-particle properties in the production of particleboard. Wood Mater. Sci. Eng..

[31] Zvirgzds K., Kirilovs E., Kukle S., Gross U. (2022). Production of Particleboard Using Various Particle Size Hemp Shives as Filler. Materials.

[32] Regmi S., Bajwa D., Igathinathane C., Nahar N. (2022). High fiber fraction DDGS—A functional filler for manufacturing low-density particleboards. Ind. Crop. Prod..

[33] Ndiwe B., Konai N., Pizzi A., Karga L., Kaoutoing M.D., Danwe R. (2022). Mechanical performance of a particleboard based on natural hardener. Wood Mater. Sci. Eng..

[34] Clad W. (1982). Die Rohdichtesenkung bei Spanplatten Eine Literaturübersicht. Holz als Roh- und Werkst..

[35] Sellers T., Miller G.D., Fuller M.J. (1993). Kenaf core as a board raw material. For. Prod. J..

[36] Meinlschmidt P., Schirp A., Dix B., Thole V., Brinker N. Agricultural residues with light parenchyma cells and expandable filler materials for the production of lightweight particleboards. Proceedings of the International Panel Products Symposium.

[37] Irle M., Barbu M.C. (2010). Wood-based panels technology. Wood-Based Panels—An Introduction for Specialists.

[38] Boruszewski P., Borysiuk P., Mamiński M., Czechowska J. (2016). Mat compression measurements during low-density particleboard manufacturing. BioResources.

[39] Subiyanto B., Takino S., Kawai S., Sasaki H. (1991). Production of thick low-density particleboard with a semicontinuous steam-injection press. Mokuzai Gakkaishi.

[40] Stosch M. (2006). Leichtbauwerkstoffe.

[41] Guo G., Rizvi G.M., Park C.B., Lin W.S. (2004). Critical processing temperature in the manufacture of fine-celled plastic/wood-fiber composite foams. J. Appl. Polym. Sci..

[42] Faruk O., Bledzki A.K., Matuana L.M. (2007). Microcellular Foamed Wood-Plastic Composites by Different Processes: A Review. Macromol. Mater. Eng..

[43] Matuana-Malanda L., Park C.B., Balatinecz J.J. Production of Microcellular Foamed PVC/Wood-Fiber Composites: Processing and Cell Morphology Relationship. Proceedings of the Society of Plastics Engineers, Annual Technical—ANTEC.

[44] Martini-Vvedensky J.E., Suh N.P., Waldman F.A. (1984). Microcellular Closed Cell Foams and the Method of Manufacture. U.S. Patent.

[45] Moslemi A.A. (1974). Particleboard Vol. 2: Technology.

[46] (1993). Wood-Based Panels—Determination of Density.

[47] (1994). Wood-Based Panels. Determination of Modulus of Elasticity in Bending and of Bending Strength..

[48] (1999). Particleboards and Fibreboards. Determination of Tensile Strength Perpendicular to the Plane of the Board.

[49] (2011). Particleboards and Fibreboards. Determination of Resistance to Axial Withdrawal of Screws.

[50] (2020). Wood Flooring and Parquet. Determination of Resistance to Indentation. Test Method.

[51] (1999). Particleboards and Fibreboards. Determination of Swelling in Thickness after Immersion in Water.

[52] Humphrey P.E. (1982). Physical Aspects of Wood Particleboard Manufacture. Ph.D. Thesis.

[53] Humphrey P.E., Bolton A.J. (1989). The hot pressing of dry-formed wood-based composites, Part II: A simulation model for heat and moisture transfer and typical results. Holzforschung.

[54] Kelly M.W. (1977). Critical Literature Review on Relationships between Processing Parameters and Physical Properties of Particleboards.

[55] Wong E.D., Zhang M., Wang Q., Kawai S. (1998). Effects of mat moisture content and press closing speed on the formation of density profile and properties of particleboard. J. Wood Sci..

[56] Wong E.D., Zhang M., Wang Q., Kawai S. (1999). Formation of the density profile and its effects on the properties of particleboard. Wood Sci. Technol..

[57] Gamage N., Setunge S. (2014). Modelling of vertical density profile of particleboard, manufactured from hardwood sawmill residue. Wood Mater. Sci. Eng..

[58] Dunky M. Particle size distribution and glue resin consumption: How to spare costs. Proceedings of the Second European Panel Products Symposium.

[59] Liri O., Kivistö A., Saarinen A. (1977). Der Einfluβ von Holzarten Spangröβ und Bindemittel auf Festigkeit und die Quellung von Spanpaltten mit höheren elastomechanischen Eigenschaften. Holzforsch. Und Holzverwert..

[60] Medved S., Resnik J. (2006). Impact of beech particle size on compaction ratio of the surface layer. Wood Res..

[61] Dziurka D., Mirski R., Dukarska D., Derkowski A. (2015). Possibility of using the expanded polystyrene and rape straw to the manufacture of lightweight particleboards. Maderas. Cienc. Y Tecnol..

[62] Mir S., Farrokhpayam S.R., Nazerian M., Mansouri H.R. (2016). Light weight particle board using expanded polystyrene. J. Wood For. Sci. Technol..

[63] Luo S., Gao L., Guo W. (2020). Effect of expanded polystyrene content and press temperature on the properties of low-density wood particleboard. Maderas. Cienc. Y Tecnol..

[64] (2014). Lightweight Particleboards—Specifications.

[65] Stosch M. (2009). Leichtbau-Werkstoffe, Technologie, Verarbaitung.

[66] Thoemen H., Irle M., Sernek M. (2010). Wood-Based Panels: An Introduction for Specialists.

[67] Roffael E., Rauch W. (1972). Influence of density on the swelling behaviour of phenolic resin bonded particle. Holz Roh Werkst..

[68] Boehme C. (1991). Thickness swelling of chip boards for furniture in view of the CEN-standardization. Holz Roh Werkst..

[69] Horvath J. (1994). Expanded Polystyrene (EPS) geofoam: An introduction to material behavior. Geotext. Geomembr..

